# Cloud-Based Multicenter Data Collection and Epidemiologic Analysis of Bisphosphonate-Related Osteonecrosis of the Jaws in a Central European Population

**DOI:** 10.3390/jcm9020426

**Published:** 2020-02-05

**Authors:** Tamás Vereb, Krisztina Boda, László Czakó, Mihály Vaszilkó, Gábor Fülöp, Gusztáv Klenk, Ágnes Janovszky, Ferenc Oberna, József Piffkó, László Seres

**Affiliations:** 1 Department of Oral and Maxillofacial Surgery, Faculty of Medicine, University of Szeged, 6725 Szeged, Hungary; agnes.janovszky@gmail.com (Á.J.); piffkojozsef@gmail.com (J.P.); seresl@yahoo.com (L.S.); 2 Department of Medical Physics and Informatics, Faculty of Medicine, University of Szeged, 6720 Szeged, Hungary; boda.krisztina@gmail.com; 3 Department of Oral and Maxillofacial Surgery, Ružinov Hospital, Comenius University Bratislava, 81499 Bratislava, Slovakia; czako@ionline.sk; 4 Department of Oro-Maxillofacial Surgery and Stomatology, Faculty of Dentistry, Semmelweis University, 1085 Budapest, Hungary; mvaszilko@gmail.com; 5 Somogy County Kaposi Mor Teaching Hospital, 7400 Kaposvar, Hungary; fulop.gabor@kmmk.hu; 6 Department of Ear-, Nose-, Throat and Oral Surgery, St. John’s Hospital, 1125 Budapest, Hungary; dr.klenk.g@gmail.com; 7 Department of Maxillofacial and Reconstructive Surgery, National Institute of Oncology, 1122 Budapest, Hungary; oberna.ferenc@gmail.com

**Keywords:** bisphosphonate, osteonecrosis of jaws, cloud-based, data collection, multicenter

## Abstract

**Objective**: Bisphosphonate-related osteonecrosis of the jaws is considered to be a rare but severe complication of bisphosphonate therapy. To understand this condition better, data collection is essential. Although the number of scientific papers about this subject is large, to date only a few multicenter reports have been published. **Study design**: We present a novel cloud-based data collection system for the evaluation of the risk factors of bisphosphonate-related osteonecrosis of the jaws. Web-based questionnaire and database have been set up and made available to voluntary researchers and clinicians in oral and maxillofacial surgery in Hungary and Slovakia. **Results**: To date, fifteen colleagues from eight maxillofacial units have joined the study. Data of 180 patients have been recorded. Collected data were statistically analysed and evaluated from an epidemiological point of view. **Conclusions**: Authors consider cloud-based multicenter data collection a useful tool that allows for real-time collaboration between users, facilitates fast data entry and analysis, and thus considerably contributes to widening our knowledge of bisphosphonate-related osteonecrosis of the jaws.

## 1. Introduction

Bisphosphonate-related osteonecrosis of the jaws (BRONJ) was first reported in 2003 by Marx [1]. The signs and symptoms of this condition are broad and include, amongst others, exposed and necrotic bone that is isolated to the jaw; pain; tooth mobility; mucosal swelling; erythema; and ulceration [2,3,4]. To distinguish BRONJ from other delayed healing conditions, in 2007 the following definition was suggested by the American Association of Oral and Maxillofacial Surgeons: ‘’patients may be considered to have BRONJ if all of the following three characteristics are present: current or previous treatment with a bisphosphonate; exposed bone in the maxillofacial region that has persisted for more than eight weeks; and no history of radiation therapy to the jaws’’ [5].

In 2014, the revised AAOMS Position Paper recommended changing the nomenclature of BRONJ to MRONJ (medication-related osteonecrosis of the jaw), and the working definition of this condition was modified to accommodate the growing number of osteonecrosis cases associated with other antiresorptive (denosumab) and antiangiogenic therapies (Table 1) [6].

The present study had been commenced before these modifications were published; therefore, in this paper nomenclature from the 2009 AAOMS Position Paper are used [7]. The pathophysiology of BRONJ is not completely clarified; several pathways are suggested that could elucidate the unique localization of the disease, including inflammation, infection, microtrauma, altered bone remodeling, soft tissue toxicity, and angiogenesis inhibition [8,9,10,11,12,13].

Most authors agree that expanding our current knowledge is the basis for effective prevention and therapeutic strategies [14,15,16]. The literature on BRONJ is enormous and growing, but most of these papers are case presentations or reports on a small series of patients. Data are not statistically significant in most of these studies due to the low number of patients included [17,18,19,20].

The number of subjects treated in a single maxillofacial or dental unit is likely insufficient to draw statistically significant conclusions. Although there are some large patient cohorts in this field, most of these have had methodological limitations, including the lack of standardized case definitions, the absence of information on patients characteristics, and the scarcity of comparable data [21,22,23,24,25,26,27,28]. Recently, some single-institution studies with a large number of patients have been published in this field [29,30,31,32]. The aim of our study was to create a multicenter data collection system that would gather demographic, clinical, and pharmacological data of a large number of patients suffering from BRONJ [33,34,35,36,37,38,39,40,41].

A cloud database allows for real-time collaboration between several users, and thus it considerably contributes to fast data entry, processing, and analysis. It eliminates the difficulties of traditional information transfer. Although this technology has been around for several years, it has not gained popularity in medical demographic studies [42] (Table 2).

## 2. Materials and Methods

In 2012, following the approval of the local Scientific and Research Ethics Committee of the Medical Research Council, an online spreadsheet-based questionnaire was set up. 

The questionnaire has been made available to cooperating maxillofacial units for nonprofit use. The participation has been voluntary. Data have been collected on gender, age on onset of the osteonecrosis, underlying and concomitant diseases, dental history, type of bisphosphonate taken, duration, frequency and route of administration, presumed trigger factors (e.g., tooth extraction, implant placement, periodontal surgery), clinical stage, location, and extent of lesions. 

The inclusion criteria of the study were determined as follows:

(1) Previous or current BP treatment regardless of drug type, dose, or route of administration;
(2) Consecutive or concomitant BP treatment with different bisphosphonate agents;
(3) Intra- or extraoral lesion following or after bisphosphonate therapy.

Patients receiving bisphosphonate treatment but without symptoms were not included in the study. Osteonecrosis cases associated with consecutive and/or concomitant combined antiresorptive and antiangiogenic therapies were not taken into consideration in this study. Patients with previous or undergoing radiation therapy of head and neck region have been excluded. Originally, staging was determined according to the Position Paper on Bisphosphonate-Related Osteonecrosis of the Jaw published by the American Association of Oral and Maxillofacial Surgeons in 2009 but later it was modified according to the 2014 AAOMS’s update [6,7].

A detailed dental and medical history was recorded with regard to previous and current chronic diseases. Clarification of triggering factors during the development of osteonecrosis has received special attention during retrospective data collection. To determine the location and extent of the osteonecrotic lesions both jaws were divided into five regions (RM: right molar, RP: right premolar, Fr: frontal, LP: left premolar, and LM: left molar). If more than one regions were affected by the necrosis, each were taken into consideration.

Statistical analysis was performed with Statistical Program for Social Sciences version 23.0 for Windows (SPSS, Chicago, IL, USA). A *p*-value of less than 0.05 was interpreted to imply statistical significance. Means and standard deviations (SD) were calculated. Unpaired student’s t-test was used for evaluation of statistical significance.

## 3. Results

To date, seven maxillofacial units from Hungary and one from Slovakia have joined the study. Altogether, 15 colleagues participated in the data collection. The data of 180 BRONJ cases have been recorded. The number of reported cases decreased from year to year 64 patients, 41 patients, 34 patients, 23 patients, and 18 patients, respectively. 

Full data were obtained for 148 (82.2%) patients. In 32 cases (17.8%), data collection was incomplete. The data on age, sex, underlying disease, method of drug administration, and staging were complete in all cases. All data were included in the statistical analysis. 

### 3.1. Sex Ratio

There was a female predominance with 58 men (32.2%) and 122 women (67.8%) affected. Male-to-female ratio was 1:2.1; this correlates well with other results (Table 3) [18,19,23,28,34].

### 3.2. Underlying Disease

The vast majority of BRONJ cases occurred in patients with malignant diseases (*n* = 140; 77.8%). Thirty four patients (18.9%) received bisphosphonate for osteoporosis; two patients (1.1%) were diagnosed with rheumatoid arthritis. In four cases (2.2%), the reasons for treatment remained unknown because of the nature of retrospective studies (Table 4).

### 3.3. Age 

The mean age at the time of the diagnosis was 66.80 years, 66.22 years in women (range 37–85 years; SD 10.29 years), and 68.02 years in men (range 42–89 years; SD 9.33 years). There was no significant difference between the ages of males and females (*p* = 0.246). Patients suffering from nonmalignant diseases (osteoporosis, rheumatoid arthritis) were generally older (*n* = 36; mean 68.57 years; range 38–84 years, SD 9.79 years) than patients with malignant disease (*n* = 140; mean 66.32 years; range 37–89 years, SD 9.43 years).

Within the malignant group renal cancer patients were generally younger (*n* = 13; mean 62.92 years; range 51–77 years, SD 8.45 years) than the rest of the group (*n* = 127; mean 66.67 years; range 37–89 years, SD 9.49 years), but the difference was not significant (*p* = 0.153). Breast cancer patients were only slightly younger (*n* = 66; mean 64.68 years; range 37–85 years, SD 10.16 years), but the age difference was statistically significant (*p* = 0.045) when compared with the rest of the group (*n* = 74; mean 67.86 years; range 48–89 years, SD 8.49 years). The mean ages of multiple myeloma and lung cancer patients were 67.62 (*n* = 16; range 57–80 years, SD 6.9 years) and 65.00 (*n* = 7; range 48–81 years, SD 10.77 years), respectively. Prostate cancer patients (*n* = 30; mean 71.57 years; range 61–89 years, SD 7.00 years) were significantly older (*p* = 0.000075) than the other malignant cases (*n* = 110; mean 64.93 years; range 37–85 years, SD 9.53 years) (Table 4). 

### 3.4. Comorbidity

Comorbidity data were complete in 162 (90%) cases. High blood pressure and/or cardiac disease was reported in 75 (46.29%) cases. Nineteen (11.72%) patients suffered from diabetes. Chronic obstructive pulmonary disease (COPD) and/or asthma were diagnosed in 6 (3.70%) cases. Concomitant renal, hepatic, and gastrointestinal diseases were reported in 11 (6.79%), 7 (4.32%), and 9 (5.56%) patients, respectively. 

### 3.5. Route of Administration and Type of Bisphosphonate

Fifty-two individuals (28.9%) were given oral bisphosphonates alone. In this group, ibandronic acid (*n* = 19; 36.6%) and alendronic acid (*n* = 18; 34.6%) were the most frequently used agents, followed by clodronic acid (*n* = 9; 17.3%) and risedronic acid (*n* = 6; 11.5%) (Table 5).

In vast majority of the cases, bisphosphonates were administered intravenously alone (*n* = 106; 58.9%) or in combination with oral drugs (*n* = 22; 12.2%). A total of 128 patients (71.1%) received intravenous bisphosphonate therapy. Intravenous zoledronic acid was associated with the highest risk of BRONJ, and 110 patients (61.1%) were treated with this drug alone or in combination with other agents (*n* = 6; 3.3%). Altogether, 116 patients (64.4% of all patients; 90.6% in the intravenously treated group) were administered intravenous zoledronic acid. (Table 6)

Results of the Pearson Chi-square test showed a statistically significant relationship (*p* = 0.023) between the severity of stages (Stage 1: mild versus Stage 2+3: serious) and the administration method (Table 7.)

### 3.6. Presumed Triggering Factors

Presumed triggering factors were reported in 167 cases. Dental extraction was the most common predisposing event (*n* = 121; 72.4%). A further six patients (3.6%) had previous dentoalveolar surgery (implant placement, periodontal surgery). Pre-existing inflammatory diseases such as periodontal and/or periapical pathology were present in 19 cases (11.4%). Denture use was thought to be the main trigger factor in 12 cases (7.2%). BRONJ was considered of spontaneous origin in nine cases (5.4%). 

### 3.7. Localization of the Osteonecrosis 

BRONJ was observed altogether in 194 jaws. In 124 patients (68.9%), only the mandible; in 42 patients (23.3%), only the maxilla; and in 14 cases (7.8%), both jaws, were affected. 

**Figure 1:**
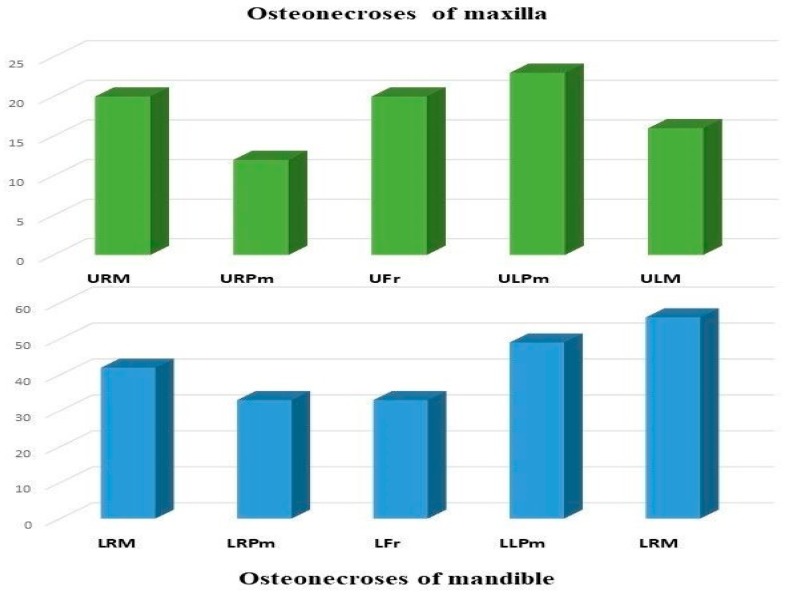
**Localizations of ONJ by regions** (Abbreviations: URM—upper right molar, URPm—upper right premolar, Ufr—upper frontal, ULPm—upper left premolar, ULM—upper left molar, LRM—lower right molar, LRPm—lower right premolar, LFr—lower frontal, LLPm—lower left premolar, LLM—lower left molar).

Altogether, 304 regions were affected by BRONJ in 180 patients, 213 regions (70.1%) in the mandible, and 91 regions (29.9%) in the maxilla. The most common sites of osteonecrosis were the molar (*n* = 98; 32.2%) and the premolar regions of the mandible (*n* = 82; 27%), followed by the upper molar (*n* = 36; 11.8%) and premolar regions (*n* = 35; 11.5%). The lower and upper front regions were affected in 33 (10.9%) and 20 (6.6%) cases, respectively (Figure 1).

### 3.8. Staging 

At the time of the first clinical examination, 36 cases (20.0%) were categorized as stage 1. The majority of the patients (*n* = 96; 53.3%) were diagnosed as stage 2. Forty-eight cases (26.7%) were classified as stage 3 with extraoral fistula; pathological fracture; and involvement of the maxillary sinus, the inferior border, or the ramus of the mandible. The underlying disease and its malignant or benign nature were determined in 176 cases. There were 36 benign cases; the distributions of stages were as follows: stage 1: 36.1% (*n* = 13), stage 2: 47.2% (*n* = 17), and stage 3 16.7% (*n* = 6), respectively. From the 140 underlying malignancies, 23 (16.4%) were classified in stage 1, 77 cases (55.0%) ranked as stage 2, and 40 cases (28.6%) belonged to stage 3. At the time of the first visit, more severe stages (2–3) occured in a higher proportion of patients with malignancies than among patients with benign conditions (stage 1: 36.1% vs. 16.4%, stage 2: 47.2% vs. 55.0%, stage 3: 16.7% vs. 28.6%). As stage worsened, the proportion of malignant cases increased significantly compared to the number of benign cases (stage 1—1:1.77, stage 2—1:4.53, and stage 3—1:6.66) A significant difference was found between the benign and malignant groups (Pearson Chi-Square test *p* = 0.026). Although much more women than men are affected by BRONJ, in stage 3 the numbers of female and male patients were almost equal (26 (54.2%) and 22 (45.8%), respectively).

## 4. Discussion

BRONJ is a relatively newly recognized condition that has generated great interest not only amongst oral and maxillofacial surgeons but also in other medical and research communities. 

Our study found female predominance among BRONJ patients (female 67.8%, male: 32.2%; male to female ratio 1:2.1), which is in line with the results of Otto and Schubert [23,34] but slightly higher than in Kos’ and Mavrokokki’s publications [18,28]. Female-to-male ratio of 8:1 was published by Pazianas in 2007 [35].

77.8% of the patients suffered from an underlying malignant disease, a proportion that closely correlated with Mavrokokki’s result, who referred to 72% of bone malignancies among their patients [28]. The mean age of the benign group (68.57 years) is not significantly higher than the age of the malignant group (66.32 years). Within the malignant group, BRONJ developed at a significantly higher age in prostate cancer patients compared to the remainder of the group. BRONJ was diagnosed at a significantly younger age in breast cancer patients compared to the rest of the malignant group. Although in our investigation there were only two rheumatoid arthritis patients (mean: 39.0 years SD: 1 years), there was still a surprisingly huge age difference when it was compared to the results of Di Fede (*n* = 18 mean: 68 years SD: 8 years) [17].

Gabbert’s examination pointed out that osteonecrosis-free survival in single bisphosphonate users was significantly longer in pamidronate-treated patients than in zoledronate or ibandronate users [27]. In our study, from the intravenous group 127 of 128 patients (99.2%) were administered zoledronate and/or ibandronate and only one patient (0.8%) was diagnosed with BRONJ following pamidronate treatment. Our results also prove that the route of administration has a significant (*p* = 0.023) association with the severity of the osteonecrosis. According to Thumbigere-Math et al., increased cumulative doses and long-term bisphosphonate treatment are the most important risk factors for osteonecrosis, but the type of bisphosphonate may also play a role in the incidence of osteonecrosis; our results confirm these findings [26].

According to the literature, the mandible is affected in 64 to 70.6%, and the maxilla is involved in 18.3 to 27%. BRONJ was present in both jaws in 9% to 11.1%. Our findings (mandible 70.1%, maxilla 23.3%, and both jaws 7.8%) correlate well with these results. There is a characteristic distribution of osteonecrosis with a predilection for the molar and premolar region in both jaws, just as it was pointed out by Otto et al. [28,34].

At the time of the diagnosis, the majority of the patients (53.3%) were categorized as stage 2; 20.0% and 26.7% were classified as stage 1 and stage 3, respectively. These findings are similar to those of Schiodt et al. (stage 1: 26%; stage 2: 58%; stage 3: 10%; unknown: 3%; resolved: 2%) [39]. Although much more women than men are affected by BRONJ, their number in stage 3 is nearly the same (26 and 22, respectively). The ratio of malignant cases to benign cases increased significantly (*p* = 0.026) as the stage worsened (stage 1—1:1.77, stage 2—1:4.53, and stage 3—1:6.66).

The evolution of cloud-based information technology has dramatically changed data collection and analysis for scientific purposes. To the best of our knowledge, our study is the first one that has collected data on BRONJ patients from multiple centers with this method. 

Despite the many advantages offered by cloud-based technology, our study also has some pitfalls. Participation was voluntary, and this probably resulted in under-reporting; therefore, our data are not informative about the incidence of BRONJ. The relatively high number of incomplete reports is surprising, but this can be explained by the fact that the online questionnaire was not filled out at the time of the patient’s examination and later data were not found in the documents. The number of patients reported in this study is high compared to other single-center or even multicenter studies, but the average number of patients reported per center per year is less than six in the 4-year study period.

The decreasing trend of the number of new patients reported per year probably reflects that voluntary researchers have lost their initial enthusiasm, but better patient management, early diagnosis, and state-of-the-art prevention techniques might also have played an important role. A sample size of 180 BRONJ cases is considered statistically significant, but data were not always sufficient to reach statistically reliable conclusions when the patients were classified into groups. More patients are needed to improve power of the study. A multicenter registry that collects systematic information on epidemiological data is essential to increase our knowledge of BRONJ. Cloud-based information collection is an ideal tool for this purpose. The online and voluntary nature of the current study may slightly diminish the accuracy of the results, but the increasing number of patients involved will improve statistical conclusions. The data collection is ongoing to improve the scientific value of the study.

## Figures and Tables

**Table 1:** Changes in the nomenclature and definition of bisphosphonate/medication related osteonecrosis of the jaws.

Definition of Bisphosphonate-Related Osteonecrosis of the Jaws (BRONJ) (AAOMS 2009)	Definition of MRONJ (AAOMS 2014)
1. Current or previous treatment with a bisphosphonate; 2. Exposed bone in the maxillofacial region that has persisted for more than eight weeks; 3. No history of radiation therapy to the jaws.	1. Current or previous treatment with antiresorptive or antiangiogenic agents; 2. Exposed bone or bone that can be probed through an intraoral or extraoral fistula(e) in the maxillofacial region that has persisted for more than eight weeks; 3. No history of radiation therapy to the jaws or obvious metastatic disease to the jaws.

(AAOMS—American Association of Oral and Maxillofacial Surgeons; MRONJ—medication related osteonecrosis of the jaw).

**Table 2:** Advantages of spreadsheet-based questionnaires.

Easy to use/apply for simple users; Allow for easy collaboration regardless of distance; (Common) database eliminates duplicate information; Increase efficiency and data consistency; Automatic backup prevents accidental data loss; Data integrity and portability are assured between platforms; Control access permissions and user restrictions; Easy retrieval and updating of data; Only the bandwidth of the network limits the speed of data transmission; Information security; The user’s interface can easily be adapted to the needs of the survey.

**Table 3:** Male–female ratio by different authors.

	Number	Female		Male	
Vereb et al.	180	122	67.8%	58	32.2%
Otto 2012	126	92	73.0%	34	27.0%
Diniz-Freitas 2012	20	19	95%	1	5%
Schubert 2011	258	175	67.8%	83	32.2%
Kos 2010	34	19	55.9%	15	44.1%
Mavrokokki 2007	114	63	55%	51	45%
Summary	732	490	66.9%	242	33.1%

**Table 4:** Age distribution based on known underlying disease (*n* = 176).

Underlying Disease		*n*	Mean	Min	Max	SD
Malignant	Breast cc.	66	64.68	37	85	10.16
	Prostate cc.	30	71.57	61	89	7.00
	Multiple myeloma	16	67.62	57	80	6.09
	Renal cc.	13	62.92	51	77	8.45
	Lung cc.	7	65.00	48	81	10.77
	Gastrointestinal cc.	5	66.80	54	80	–
	Other	3	62.00	53	75	–
	Total malignant	140	66.32	37	89	9.43
Benign	Osteoporosis	34	70.31	42	84	9.79
	Rheumatoid arthritis	2	39	38	40	–
	Total benign	36	68.57	38	84	–
Summary		176	66.80	37	89	–

**Table 5:** BRONJ cases caused by oral bisphosphonates.

Oral Bisphosphonate	Benign Diseases	Malignant Diseases	Summary
aledronate	17	1	18
ibadronate	2	17	19
clodronate	4	5	9
risedronate	6	–	6
Summary	29	23	52

**Table 6:** Development of osteonecrosis depending on the type of intravenous bisphosphonate and the number of infusions administered.

Number of Bisphosphonate Infusions	Zoledronate (Combined)	Zoledronate (Single)	Ibandronate	Pamidronate
1–6 x	–	29	–	–
7–12 x	1	17	5	–
13–18 x	–	11	1	–
19–24 x	3	12	3	–
25–30 x	1	6	1	–
31–36 x	–	7	–	–
37–42 x	–	2	–	–
43–48 x	–	6	1	–
>48 x	1	20	–	1
Summary	6	110	11	1

**Table 7:** The correlation between the severity of osteonecrosis of the jaws (ONJ) and the route of administration.

	Staging			Total
	Stage 1	Stage 2	Stage 3	
Intravenous	14	57	35	106
	13.2%	53.8%	33.0%	100.0%
Both	7	11	4	22
	31.8%	50.0%	18.2%	100.0%
Oral	15	28	9	52
	28.8%	53.8%	17.3%	100.0%
Total	36	96	48	180
	20.0%	53.3%	26.7%	100.0%
